# From Polarity to Plurality: Perceptions of COVID‐19 and Policy Measures in England and Scotland

**DOI:** 10.1111/hex.14112

**Published:** 2024-06-14

**Authors:** Hinpetch Daungsupawong, Viroj Wiwanitkit

**Affiliations:** ^1^ Private Academic Consultant Phonhong Lao People's Democratic Republic; ^2^ Department of Research Analytics, Saveetha Dental College and Hospitals, Saveetha Institute of Medical and Technical Sciences Saveetha University Chennai TN India

Dear Editor,

We would like to comment on ‘From polarity to plurality: Perceptions of COVID‐19 and policy measures in England and Scotland’ [1]. The purpose of this study was to look into different viewpoints regarding the COVID‐19 epidemic and the UK and Scottish governments' responses to stop its spread. Perspectives from people with different experiences of the pandemic, including critical workers, public health officials and furloughed employees, were gathered using the Q technique. After participants ranked COVID‐19‐related terms based on their points of view, factor analysis was used to determine the various viewpoints. The goal of the study was to offer information that might guide various levels of pandemic response in the future.

The study's findings, which were bolstered by participant stories and statistical analysis, presented four different viewpoints on the COVID‐19 epidemic. These viewpoints included opinions on crisis management, responsibility for leadership, feelings of dread and rage at laws and public reactions, and injustices that were brought to light. In addition to highlighting several important concerns with the response operations, the study offered insightful information about the various viewpoints on the epidemic.

The small sample size and possible bias in the participant selection are two possible weaknesses of the study. A larger and more varied sample could have helped the study capture a greater variety of viewpoints. Furthermore, there can be a barrier to obtaining subtle and complex viewpoints on a topic as complex as the COVID‐19 epidemic when using the Q methodology.

Potential avenues for further research may entail broadening the scope of the study to encompass viewpoints from a more extensive and heterogeneous group of participants residing in various geographical areas. Using a longitudinal approach could also shed light on how attitudes toward the pandemic change over time. Additionally, investigating the pandemic's effects on various demographic groups and oppressed communities may provide important new light on the injustices that the crisis has brought to light. All things considered, this work establishes the groundwork for future investigations into comprehending and resolving the problems caused by pandemics.

## Author Contributions

**Hinpetch Daungsupawong:** 50% ideas, writing, analysing, approval. **Viroj Wiwanitkit:** 50% ideas, supervision, approval.

## Conflicts of Interest

The authors declare no conflicts of interest.

## Data Availability

The authors have nothing to report.

## References

[1] J. Rendall , N. McHugh , R. Baker , H. Mason , and O. Biosca , “From Polarity to Plurality: Perceptions of COVID‐19 and Policy Measures in England and Scotland,” Health Expectations 27, no. 3 (June 2024): e14069. 10.1111/hex.14069. 38733243 PMC11087883

